# Those Were Happy Days

**Published:** 1916-12

**Authors:** 


### Those Were Happy Days:

When the doctor could shake his head sadly and murmur that there was nothing to be done—and do so with a clear conscience.

When almost any well informed physician could, in his spare time, do research work that deserved respectful consideration.

When almost any group of seven physicians could hire a building and start a medical college that would pay expenses and even salaries.

When social position, a little money and an aggressive disposition would not only secure a large practice but professional prestige.

When almost any practitioner could speak patronizingly and sceptically of germs and bacteriologists.

When we could sit on the branch of preventable diseases, saw away on it, on the side nearer the trunk, have a sense of personal righteousness and, at the same time, the comfortable economic assurance that it would be a long long time before we produced any weakness of our support.

When the quack travelled with a tent, a band and a ventriloquist's outfit, instead of having a college degree and a better X-ray equipment than our own.

When a doctor could make more of an impression with a $350 horse and buggy than he can now with an $800 automobile.

When the boy who restrained his impatience to study medicine till he had done three years' high school work, could feel that he had voluntarily made a heavy sacrifice to the cause of educational preparedness.

When interneships were frankly stated to go, like kisses, by favor and not according to merit.

When no one accused professional organization of being a vast medical trust controlled by a few but when each little, local society was an independent camp and often in a state of internal discord.

